# To spray or target mosquitoes another way: focused entomological intelligence guides the implementation of indoor residual spraying in southern Mozambique

**DOI:** 10.1186/s12936-022-04233-3

**Published:** 2022-07-10

**Authors:** Celso Alafo, Helena Martí-Soler, Mara Máquina, Arlindo Malheia, Ayesha S. Aswat, Lizette L. Koekemoer, James Colborn, Neil F. Lobo, Allison Tatarsky, Yasmin A. Williams, Dulcisária Marrenjo, Nelson Cuamba, Regina Rabinovich, Pedro Alonso, Pedro Aide, Francisco Saúte, Krijn P. Paaijmans

**Affiliations:** 1 Centro de Investigação Em Saúde de Manhiça, Fundação Manhiça, Maputo, Mozambique; 2 ISGlobal, Barcelona, Spain; 3 WITS Research Institute for Malaria, Faculty of Health Sciences, University of the Witwatersrand, & National Institute for Communicable Diseases, Johannesburg, South Africa; 4 Clinton Health Access Initiative, Boston, USA; 5 Eck Institute for Global Health, University of Notre Dame, Notre Dame, IN USA; 6 Malaria Elimination Initiative, Institute of Global Health Sciences, University of California San Francisco, San Francisco, CA USA; 7 Programa Nacional de Controlo da Malária, Ministério da Saúde, Maputo, Mozambique; 8 PMI VectorLink Project, Abt Associates Inc, Maputo, Mozambique; 9 Harvard T.H. Chan School of Public Health, Boston, MA USA; 10 Instituto Nacional da Saúde, Ministério da Saúde, Maputo, Mozambique; 11 Center for Evolution and Medicine, School of Life Sciences, Arizona State University, Tempe, AZ USA; 12 The Biodesign Center for Immunotherapy, Vaccines and Virotherapy, Arizona State University, Tempe, AZ USA; 13 Simon A. Levin Mathematical, Computational and Modeling Sciences Center, Arizona State University, Tempe, AZ USA

**Keywords:** Malaria elimination, Entomological indicators, *Anopheles* surveillance, Vector control, Implementation science

## Abstract

**Background:**

To eliminate malaria in southern Mozambique, the National Malaria Control Programme and its partners are scaling up indoor residual spraying (IRS) activities in two provinces, Gaza and Inhambane. An entomological surveillance planning tool (ESPT) was used to answer the programmatic question of whether IRS would be effective in target geographies, given limited information on local vector bionomics.

**Methods:**

Entomological intelligence was collected in six sentinel sites at the end of the rainy season (April–May 2018) and the beginning of the dry season (June–July 2018). The primary objective was to provide an ‘entomological snapshot’ by collecting question-based, timely and high-quality data within one single week in each location. Host-seeking behaviour (both indoors and outdoors) was monitored by human-baited tent traps. Indoor resting behaviour was quantified by pyrethrum spray catches and window exit traps.

**Results:**

Five different species or species groups were identified: *Anopheles funestus *sensu lato* (s.l.)* (66.0%), *Anopheles gambiae s.l.* (14.0%), *Anopheles pharoensis* (1.4%), *Anopheles tenebrosus* (14.1%) and *Anopheles ziemanni* (4.5%). *Anopheles funestus *sensu stricto* (s.s.)* was the major vector among its sibling species, and 1.9% were positive for *Plasmodium falciparum* infections. *Anopheles arabiensis* was the most abundant vector species within the *An. gambiae* complex, but none tested positive for *P. falciparum* infections. Some *An. tenebrosus* were positive for *P. falciparum* (1.3%). When evaluating behaviours that impact IRS efficacy, i.e. endophily, the known primary vector *An. funestus s.s.*, was found to rest indoors—demonstrating at least part of its population will be impacted by the intervention if insecticides are selected to which this vector is susceptible. However, other vector species, including *An. gambiae s.l*., *An. tenebrosus*, *An. pharoensis* and *An. ziemanni*, showed exophilic and exophagic behaviours in several of the districts surveilled.

**Conclusion:**

The targeted approach to entomological surveillance was successful in collecting question-based entomological intelligence to inform decision-making about the use of IRS in specific districts. Endophilic *An. funestus s.s.* was documented as being the most prevalent and primary malaria vector suggesting that IRS can reduce malaria transmission, but the presence of other vector species both indoors and outdoors suggests that alternative vector control interventions that target these gaps in protection may increase the impact of vector control in southern Mozambique.

**Supplementary Information:**

The online version contains supplementary material available at 10.1186/s12936-022-04233-3.

## Background

Mozambique aims to eliminate malaria in Maputo city and Maputo province, and to accelerate towards elimination (i.e. test positivity rates below 5%) in the other two southern provinces (Gaza and Inhambane) by 2025. Given porous borders with neighbouring malaria endemic countries, in-country interventions alone are not sufficient to eliminate malaria [1] and cross-border and regional collaborative efforts are needed [2, 3]. The government of Mozambique has worked closely with the governments in South Africa and Eswatini to reduce the malaria burden since 1991, when the Lubombo Spatial Development Initiative (LSDI) was initiated. This initiative led to a significant reduction in the malaria burden in border regions between 1999 and 2005 [4, 5], after the scale-up of indoor residual spraying (IRS), alongside the implementation of effective diagnostics and treatment with rapid diagnostic tests (RDTs) and artemisinin-based combination therapy (ACT), respectively, in southern Mozambique [6, 7]. In Maputo province, *Plasmodium falciparum* malaria prevalence in children 2–14 years decreased from 65–70% to 4–33% during this period, and malaria case reductions of 95%, 96% and 78% were reported in Eswatini, KwaZulu-Natal (South Africa) and Mpumalanga (South Africa), respectively [5]. In 2014, almost ten years after the end of LSDI, the MOSASWA (Mozambique, South Africa and Eswatini) initiative was launched. The aim was to significantly reduce malaria sub-nationally in southern Mozambique, and to transition from (i) malaria pre-elimination to elimination in Eswatini and South Africa, and (ii) from control to pre-elimination in southern Mozambique [7]. This collaboration is evidence of the national and regional commitment to accelerate progress towards malaria elimination, as set forward by WHO’s global technical strategy for malaria elimination [8].

As part of its National Strategic Plan (NSP), Mozambique has laid out an evidence-based programme based on epidemiologically impactful interventions [9], with vector control being a core component. Frontline vector control tools include insecticide-treated nets (ITNs) or long-lasting insecticidal nets (LLINs) and IRS. The aim is to achieve universal coverage of LLINs (defined as one insecticide-treated net for every two persons in a household [10]), with nets being distributed country-wide every three years and continuously provided to pregnant women attending antenatal care [11, 12]. IRS remains an important vector control intervention [7, 9, 13, 14] and has been implemented in Zambézia province (central Mozambique) since 2007 [14], and continues to be implemented in Maputo province by the National Malaria Control Programme (NMCP) after the LSDI ended. As IRS reduced malaria in Maputo province at the beginning of this century [5], it was scaled up to cover parts of Gaza and Inhambane provinces as well.

With renewed investments by Global Fund and partners in MOSASWA, it is envisioned that an increased number of structures will be sprayed during IRS campaigns in Gaza and Inhambane provinces over the next few years to move these provinces from the control to the pre-elimination stage. The efficacy of IRS depends largely on local vector bionomics, which include important entomological indicators such as their time and place of biting and resting, in addition to their insecticide susceptibility status. This requires entomological intelligence that provides timely, informative and actionable data. Whilst the NMCP is rapidly increasing its capacity in entomology at national and regional levels, vector densities are currently only assessed through pyrethrum spray catches (PSC) in most provinces (except for areas where President’s Malaria Initiative is active, and more recently in Maputo province). This method allows the programme to monitor the number of indoor resting mosquitoes at the time of collection, but fails to capture vectors that (i) bite but do not rest indoors, (ii) bite and leave the house before the time of collection, and (iii) bite and/or rest outdoors.

To address these important gaps in understanding the key local vector characteristics and guide decision-making on vector control, discussions were initiated in 2018 on how to answer key programmatic questions through a collection of entomological intelligence. The Entomological Surveillance Planning Tool (ESPT), an operational planning and decision-support tool that supports more tailored and targeted vector control, was adapted to the Mozambique context for entomological surveillance planning and selection of appropriate entomological indicators and sampling methods to help answer the programmatic questions about IRS targeting, among others [15].

Several districts in Gaza and Inhambane provinces were selected by the NMCP and partners to pilot additional surveillance methods and approaches to answer one specific programmatic question: ‘Will indoor residual spraying be effective in currently untargeted areas in Gaza and Inhambane provinces?’ A secondary objective of the pilot was to assess if malaria transmission is most likely to occur indoors or outdoors, and to see how reliable PSC, the entomological surveillance tool primarily used by the country to assess vector densities, is in evaluating malaria vector species densities and composition. Entomological intelligence was collected using a ‘snapshot entomological surveillance’ (SnES) approach and the ESPT [15]. This approach, its results and recommendations are outlined in this paper.

## Methods

### Study sites

**Fig. 1:**
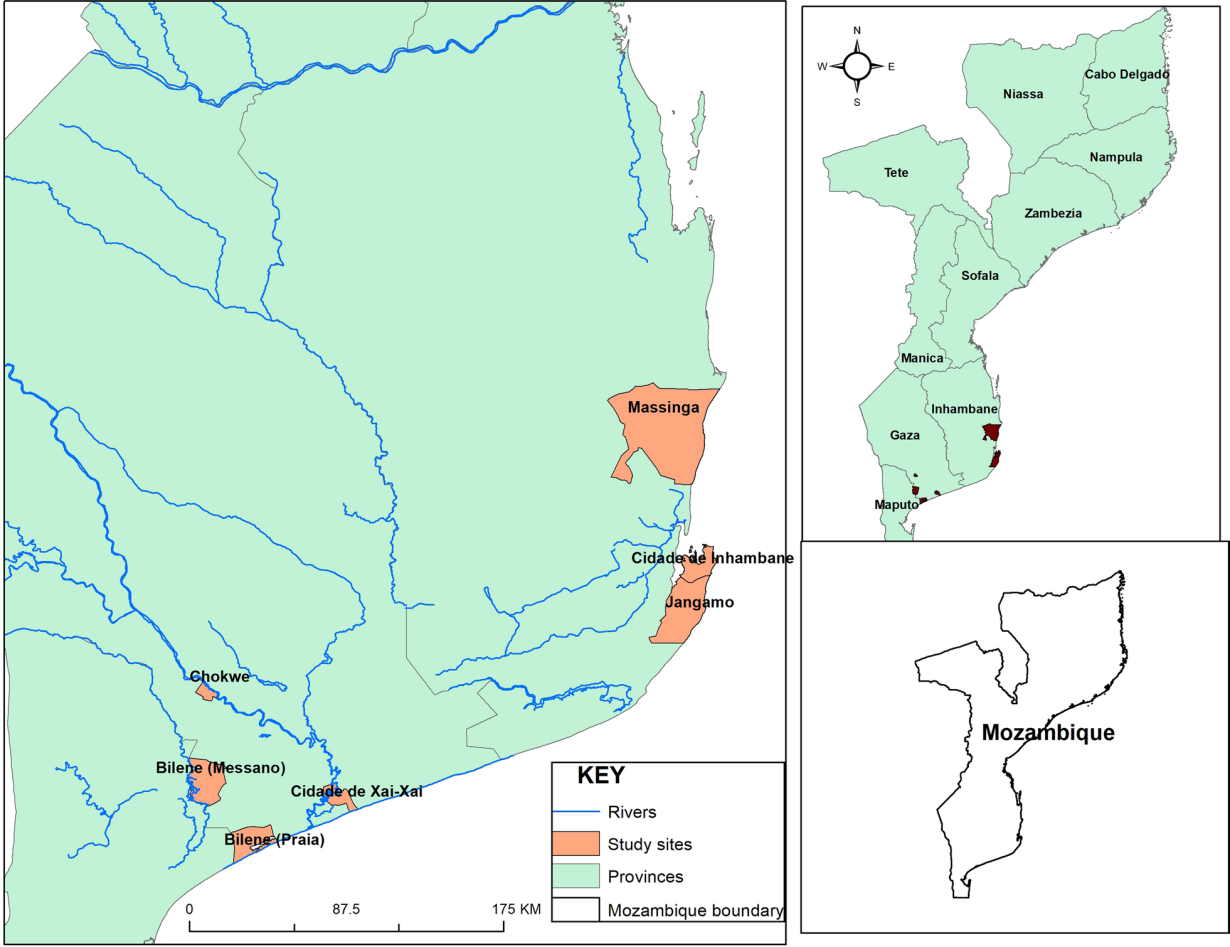
Map showing study sites in Gaza and Inhambane provinces, southern Mozambique

The study was conducted at the end of the rainy season (April–May 2018) and the beginning of the dry season (June–July 2018) in Gaza and Inhambane provinces, southern Mozambique. These are two of the most malarious provinces in southern Mozambique with prevalence rates (in children under 5) of 17% and 35%, respectively, in 2018 [16]. Gaza province is located north of Maputo Province and west of Inhambane Province, and borders with South Africa and Zimbabwe (Fig. 1). Inhambane province borders with Sofala and Manica provinces, Mozambique. Both provinces are connected with the Indian Ocean in the east. Surveillance was set up in three districts of Gaza (Bilene, Chokwe and cidade de Xai-Xai) and three districts of Inhambane provinces (cidade de Inhambane, Jangamo and Massinga). Sites were selected by the NMCP and partners based on malaria incidence and plans to expand IRS activities to those areas in 2019 and beyond.

### Snapshot entomological surveillance (SnES) and house selection

The rationale behind the SnES approach described below was to generate high-quality data on relevant vector characteristics in a short period of time to help inform an operationally feasible implementation strategy. Each district had entomological collections performed twice, with approximately eight weeks between sampling periods (Additional file 1 shows the dates each sentinel site was visited). Each sampling period was five days (four nights and/or mornings of collections). A total of 24 sentinel, unsprayed houses were selected in each site, equally divided over two different neighbourhoods (or bairros, an administrative subdivision within villages or towns). Twelve houses were selected to assess *Anopheles* biting behaviours (indoor versus outdoors); the other 12 were used to assess mosquito resting behaviours. The first selected house was typically the home of the bairro leader; the other houses were selected as follows: following the roads in all directions from the first house, every 3rd household was visited. If all inclusion criteria were fulfilled (i.e. homeowner present, homeowner agrees to participate, adequate and safe space for placing the tent trap indoors and outdoors, adult male volunteer present to sleep in the tent trap, and/or window present in the bedroom for placement of exit trap), the household was enrolled in the study. If not, the neighbouring household was visited.

### Mosquito biting behaviour

**Fig. 2:**
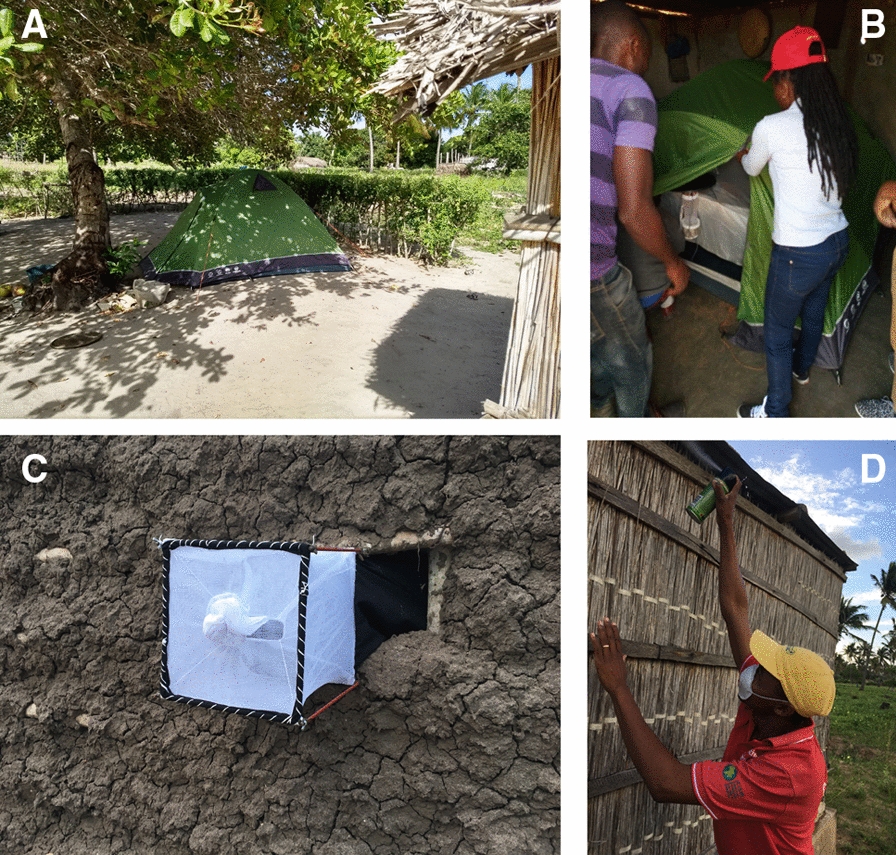
Various mosquito collection tools used in the study. **A** Human-baited Tent Trap outdoors; **B** Human-baited Tent Trap indoors, **C** window exit trap, **D** Pyrethreum Spray Catch, a fieldworker spraying the eaves (from the outside)

Indoor and outdoor biting preference were assessed with human-baited tent traps (HBTTs, Fig. 2A and B). A CDC Miniature Light Trap (CDC-LT) (Model 512, John W Hock, USA) was hung inside a standard camping tent (Natural Instincts Highveld 3 Tent; L × W × H: 2,1 × 2,1 × 1,3 m) in the space between the inner and outer tent. This tent type was selected as mosquitoes can enter the outer tent from 360° just above the ground. One adult (> 18 years old) male volunteer, a member of the household where the tent was placed, stayed inside the tent from sunset to sunrise (17:00–6:00). This person was protected from mosquito bites by the inner tent, which mosquitoes could not enter. For indoor mosquito collections (Fig. 2B) the tent plus its occupant was placed in a room where no other persons were sleeping (e.g. living room space); for outdoor mosquito collections, the tent was placed on the compound, preferably under a tree (Fig. 2A). Mosquito collections were performed for two consecutive nights in each household: the first night the tent was placed indoors; the second night (after collecting all mosquitoes) outdoors (or vice versa). This resulted in 12 trapping nights indoors and 12 outdoors every week.

### Mosquito resting behaviour

To study mosquito house resting and exiting behaviour during the night, one window exit trap (WT) was attached to the window of a bedroom where at least one person would sleep that night. Additional windows in the same bedroom (uncommon) were either closed or sealed with a piece of black cloth. The WT consisted of a metal frame (30 × 30 × 30 cm) covered with untreated netting and with a funnel opening that allowed mosquitoes to fly in but not out (Fig. 2C). WTs were implemented just before sunset and the mosquitoes were collected the following morning after sunrise prior to the PSC, which was conducted in the same room.

PSCs were conducted during the early morning (6–10 am) in a single room where at least one person slept the night before (the actual number of persons that slept in the room that night was recorded the next day, see below). Household members were asked to stay outside, the floor was covered with white sheets and all mosquito escape routes (windows, openings in the wall) were closed prior to simultaneously spaying the walls (indoors) and eaves (outdoor) with a commercially available pyrethroid—piperonyl butoxide (PBO) combination (Baygon, S.C. Johnson & Son, USA; Fig. 2D). The room was left alone for fifteen minutes, after which the white sheets were moved outdoors and all knocked-down *Anopheles* mosquitoes collected. Both PSC and WT mosquitoes were collected from 12 houses per week for a total of 12 trapping events per sampling period.

### Insecticide susceptibility tests

Wild *Anopheles* mosquitoes were collected indoors from houses other than our sentinel houses (outlined above) during the early morning (06:00–10:00) using mouth or mechanical aspirators (Improved Prokopack Aspirator Model 1419, John W. Hock, USA) and their insecticide susceptibility was assessed the same day using WHO tube bioassay materials and procedures [17]. A maximum of 25 female mosquitoes were introduced into each holding tube for 1 h after which they were exposed to 0.25% pirimiphos-methyl (prioritized, given the envisioned IRS product Actellic 300CS, Syngenta, Switzerland) or 0.05% deltamethrin (a pyrethroid, which is the chemical class used in LLINs), as well as to their respective controls (olive oil and silicone oil, respectively). After 60 min the mosquitoes were transferred back to holding tubes with access to 10% sugar solution, and mortality was recorded 24 h post-exposure. Susceptibility tests were conducted at 25 ± 2 °C and 63 ± 8% RH (ambient conditions in guesthouses where the team was staying for the night), after which mosquitoes were identified morphologically.

### Laboratory analysis

*Anopheles* mosquitoes were identified to species or species groups using a stereomicroscope and dichotomous key of Gillies and Coetzee [18]. Individuals belonging to the *An. gambiae* or *An. funestus* complexes were identified to species by polymerase chain reaction (PCR) [19, 20]. The presence of *P. falciparum* and *Plasmodium vivax* (Pv210 and Pv247) parasite circumsporozoite proteins was assessed using the “sandwich” ELISA technique on the head and thorax of each individual [21]. When positive, samples were analysed again by ELISA after a boiling step to eliminate false positives [22]. Mosquitoes that were positive twice were reported as being infectious.

### Data quality and analysis

A questionnaire (using ODK software—version ODK_Collect_v1.4.4) was used to collect basic household information such as geo-coordinates, name of the head of the household (to ensure the same house was visited every visit) and type of house. The form was designed to ensure data quality by asking daily questions to assess (i) if and how many people slept in the tent or room with the trap the previous night, (ii) if the trap was still operational after a night’s collection (fan and light still on), (iii) if they cooked or used fire inside the room during the study period and (iv) if vector control tools were used.

To answer the principal programmatic question ‘*Will indoor residual spraying be effective in currently untargeted areas in Gaza and Inhambane provinces?*’ the mean number of indoor resting mosquitoes per person (for the room where PSC was conducted) is given for each geography (district), season (rainy versus dry) and *Anopheles* species. In addition, the mean number of mosquitoes per person that exited during the night (collected in the WT, from the room where PSC was conducted) is provided to estimate the additional number of mosquitoes that may have entered houses. Note that though distribution of the number of mosquitoes is not normally distributed (using Shapiro Wilk test: HBTT data: W = 0.23221, *p* < 2.2e−16; PSC and WT data: W = 0.29945, p < 2.2e−16), both the mean and the standard deviation have been reported (Additional file 2: Table S2 and Additional file 3: Table S3) due to the number of zeros in the data (median and 75th-percentile were frequently 0).

To answer the secondary study question ‘*Is malaria transmission more likely to occur indoors or outdoors in Gaza and Inhambane provinces?*’ the number of indoor and outdoor feeding mosquitoes (collected through the HBTT, presented as the mean number of biting mosquitoes per person) is estimated for each geography (district), season (rainy versus dry) and *Anopheles* species. Due to arboviral diseases transmission in Mozambique [23, 24], no comparison against human landing catches (HLC), the current gold standard methodology to assess human biting rates, was performed. Hence, HBTT collections are taken as a proxy for HLC (or biting).

By subsequently matching the three datasets (resting, exiting and biting behaviours) the proportion of mosquitoes that IRS effectively targets is calculated for each geography (district), season (rainy versus dry) and *Anopheles* species as follows:

$${{\text{Minimum\, estimated\, IRS\, effectiveness}}} ={\text{Mean}}\, \# {\text{of\,mosquitoes\,resting\, indoors}}/\left[ {\text{Mean}}\, \# {\text{{of\,mosquitoes\,biting\, indoors}}} + {\text{Mean}}\,\# {\text{of\, mosquitoes\,biting\,outdoors}}\right],$$

expressed as mosquitoes/person. This indicator described the proportion of mosquitoes that are found resting on indoor surfaces (potentially sprayed, and could be killed by IRS) out of all mosquitoes that were observed to bite in the community (both indoors and outdoors, as a measure of vector density). Values > 1 indicate that more mosquitoes are found resting than biting; values < 1 that more mosquitoes are found biting than resting.

By providing the (mean # indoor biting):(mean # outdoor biting) ratio additionally allows for endo- and/or exophagic behaviours to be assessed. Values > 1 indicative of more frequent indoor biting and values < 1 indicative of more frequent outdoor biting.

$${{\text{Maximum\ estimated\ IRS\ effectiveness}}}= \left[{\text{Mean}}\ \# {\text{of \ mosquitoes\ resting\ indoors}} + {\text{Mean}}\, \# {\text{of\ mosquitoes \ exiting}} ]/ {\text{Mean}}\ \# \ {\text{of \ mosquitoes\ biting\ indoors}} + {\text{Mean}}\ \# {\text{of \ mosquitoes\ biting\ outdoors}}\right],$$

expressed as mosquitoes/person, with the assumption that all mosquitoes in the WT rested indoors before leaving the room. This indicator describes the proportion of mosquitoes that may have rested on surfaces (and could be killed by IRS) out of all mosquitoes that were observed to bite in the community (both indoors and outdoors) as a measure of vector density.

Insecticide susceptibility was assessed by quantifying mosquito mortality 24 h post-exposure to insecticide-treated or control papers as the percentage of mosquitoes that died out of the total number of mosquitoes exposed. When control mortality was higher than 20%, the bioassay was discarded, when it was between 5 and 20% the mortality of the exposed mosquitoes was corrected using Abbott’s formula [17]. Resistance status was defined according to WHO guidelines: susceptibility (mortality 98–100%); confirmed resistance (mortality below 90%) and suspected resistance (mortality 90–97%) [17]**.**

All data were analysed using R version 4.1.0 [25].

## Results

### Data used in the analysis

A total of 1299 (out of 1357) mosquito specimens from Gaza and Inhambane provinces were used for analysis. Fifty-eight mosquitoes were excluded because of an (*i*) unwanted behaviour of study participant(s) (cooking in the bedroom prior to our arrival, *n* = 21), (*ii*) mismatch between household ID and mosquito ID (i.e. could not trace the mosquito back to its origin of collection, *n* = 17), (*iii*) faulty equipment (fan and/or battery of the trap not working, *n* = 11) and (*iv*) erroneously stored sample (non-*Anopheles* ssp. or empty tube, *n* = 9).

### Vector species composition and malaria infection rates

A total of 1285 anopheline samples were identified morphologically. Five different species/species groups were observed in Gaza and Inhambane provinces: *An. funestus s.l.* (66.0% *n* = 848), *An. gambiae s.l.* (14.0% *n* = 180), *An. tenebrosus* (14.1% *n* = 181), *An. ziemanni* (4.5% *n* = 58) and *An. pharoensis* (1.4% *n* = 18). Fourteen mosquitoes could not be identified morphologically due to damage to the mosquitoes.

Molecular identification by PCR on a randomly selected subset of mosquitoes was performed on *An. funestus* (n = 688) and *An. gambiae* (n = 172) complex mosquitoes. Two hundred individuals could not be identified to species molecularly (*An. funestus s.l.*, *n* = 192; *An. gambiae s.l., n* = 8, see discussion). The most common member of the *An. funestus* group was *An. funestus s.s.* (98.6%, *n* = 489), which was present in all six districts. Other members included *Anopheles leesoni* (C. Inhambane, *n* = 1; Jangamo, *n* = 3), *Anopheles parensis* (Chokwe, *n* = 2) and *Anopheles rivulorum* (Chokwe, *n* = 1). *Anopheles arabiensis* was the most common member (69.2%) within the *An. gambiae* complex (Bilene, *n* = 2; Chokwe, *n* = 88; Jangamo, *n* = 28; C. Inhambane, *n* = 1), followed by *Anopheles merus* (23.8%; Bilene, *n* = 2; Chokwe, *n* = 31; Jangamo, *n* = 1; cidade de Inhambane, *n* = 7) and *Anopheles quadriannulatus* (7.0%; Bilene, *n* = 3; Chokwe, *n* = 6; cidade de Xai-Xai, *n* = 1; C. Inhambane, *n* = 2).

Out of the 935 *Anopheles* specimens analysed for the presence of *P. falciparum* and *P. vivax*, nine *An. funestus s.s.*, two *An. tenebrosus* and one *An. funestus s.l.* (identified by microscopy, but not identified to species by PCR) were positive for *P. falciparum* (Table 1). This translates to an overall infection rate of 1.9% for *An. funestus s.s*. (9 out of 481 individuals tested), 1.3% for *An. tenebrosus* (2/160) and 10% for *An. funestus s.l.* (1 out of 43) across the two sampling periods, four sampling methods and six districts. No *An. ziemanni* (*n* = 53), *An. pharoensis* (*n* = 17), *An. arabiensis* (*n* = 118), *An. merus* (*n* = 37), *An. quadriannulatus* (*n* = 12), *An. parensis* (*n* = 2) and *An. leesoni* (*n* = 4) were found to be infected with *P. falciparum*. No mosquito tested positive for *P. vivax*.

**Table 1:** Detection of *Plasmodium falciparum* in *Anopheles* species that were collected in Gaza and Inhambane province (southern Mozambique) in 2018

Species	District	Trapping method	Season	# ELISA + (total)	IR (%)
*An. tenebrosus*	Cidade de Xai-Xai	HBTT- indoor	Rainy	1 (39)	2.6
	Chokwe	WT	Rainy	1 (15)	6.7
*An. funestus* s.s	Jangamo	HBTT- outdoor	Rainy	1 (51)	2
	Cidade de inhambane	HBTT- indoor	Rainy	1 (20)	5
	Bilene	PSC	Dry	1 (68)	1.5
	Bilene	WT	Dry	1 (87)	1.1
	Bilene	HBTT- indoor	Dry	1 (62)	1.6
	Massinga	PSC	Rainy	1 (36)	2.8
	Massinga	WT	Dry	1 (10)	10
	Massinga	HBTT- indoor	Rainy	3 (28)	10.7
*An. funestus* s.l	Massinga	HBTT- indoor	Dry	1 (28)	3.6

*IR* infection rate

### Anopheles bionomics and estimated IRS impact

Below the estimated efficacy of IRS is presented by geography (Gaza province: cidade de Xai-Xai, Bilene and Chokwe districts; Inhambane province: cidade de Inhambane, Jangamo and Massinga districts). Detailed species-specific bionomic data is shown in the figures and supplementary tables.

### Bilene district

**Fig. 3:**
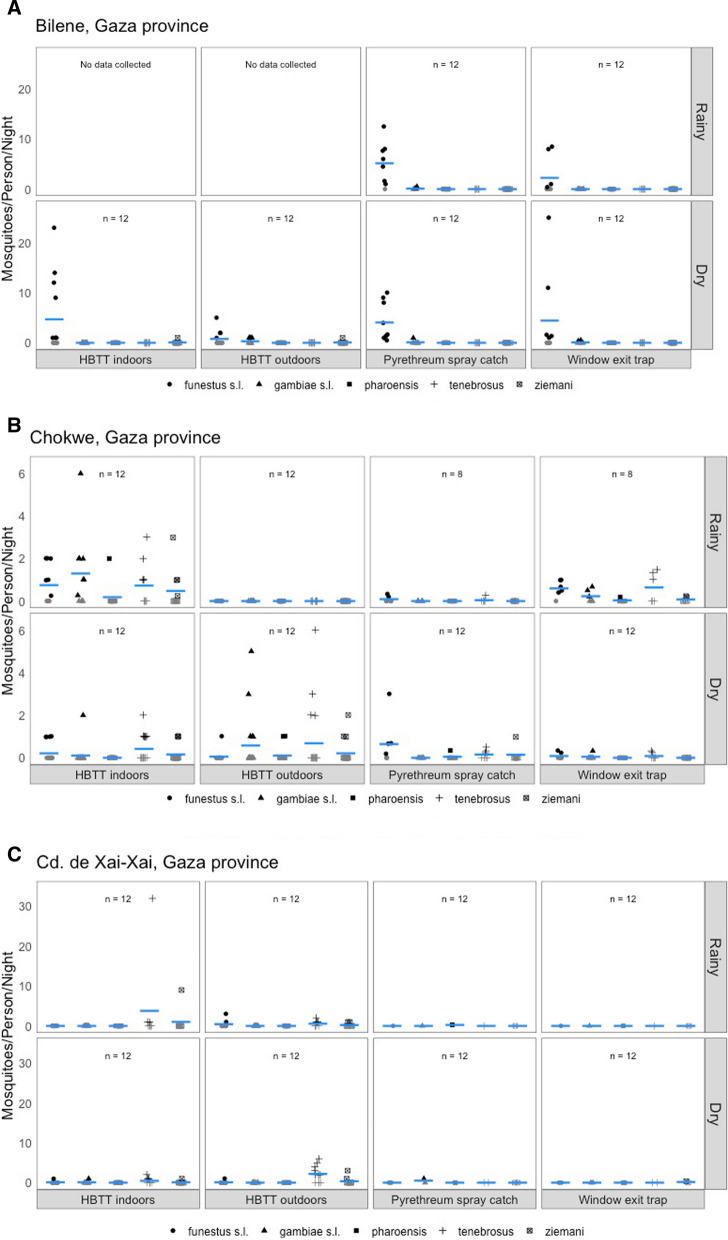

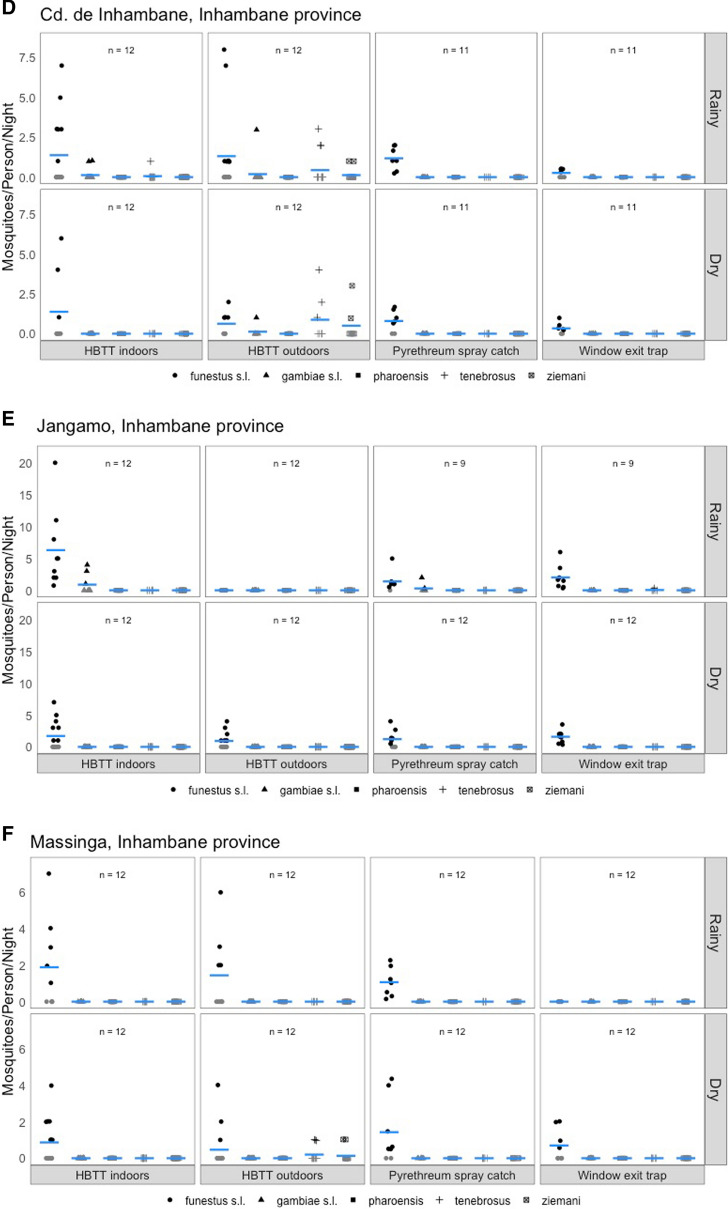
Anopheles biting, resting, and exiting behaviors in southern Mozambique during the rainy and dry season of 2018. **A-C** Gaza Province (**A**: Bilene, **B**: Chokwe, cidade de Xai Xai), **D-F** Inhambane Province (**D**: cidade de Inhambane, **E**: Jangamo, **F**: Massinga). All indicators are expressed as the mean number of mosquitoes per person

Data on mosquito biting behaviour were only collected during the dry season in Bilene, due to logistical challenges at the start of the pilot. The minimum estimated IRS efficacy based on recorded vector bionomics during this period is higher for *An. funestus s.l.* (0.74; Table 2) than for *An. gambiae s.l.* (0.35), given the high numbers of indoor resting *An. funestus s.l.* individuals compared to the numbers found biting indoors and outdoors (Fig. 3A). This species was also six times more likely to feed indoors than outdoors, whereas *An. gambiae s.l.* was only found biting outdoors. The maximum estimated IRS efficacy, considering the numbers collected in the window exit traps as well, more than doubled for *An. funestus s.l.*, but increased minimally for *An. gambiae s.l.* (from 0.36 to 0.65). Of note are two *An. ziemanni* individuals that were found biting (one indoors; one outdoors), whereas this species was not found resting inside or exiting houses.

**Table 2:** Indoor residual spraying efficacy and indoor to outdoor biting ratio shown for each geography (district), season (rainy versus dry) and anopheline species

	Rainy season			Dry season		
	Indoor:outdoor biting ratio^a^	Minimum IRS efficacy	Maximum IRS efficacy	Indoor:outdoor biting ratio^a^	Minimum IRS efficacy	Maximum IRS efficacy
Bilene (Gaza)						
* An. funestus s.l*	ND	ND	ND	**4.69**:0.77	0.74	1.56
* An. gambiae s.l*	ND	ND	ND	0:**0.31**	0.35	0.65
* An. ziemanni*	ND	ND	ND	0.08:0.08	0^b^	0^b^
Chokwe (Gaza)						
* An. funestus s.l*	**0.75**:0	0.12	0.91	**0.21**:0.05	2.50	2.81
* An. gambiae s.l*	**1.30**:0	0	0.18	0.11:**0.58**	0	0.06
* An. tenebrosus*	**0.73**:0	0.05	0.93	0.42:**0.68**	0.14	0.21
* An. pharoensis*	**0.18**:0	0	0.17	0:**0.11**	0.36	0.36
* An. ziemanni*	**0.48**:0	0	0.15	0.16:**0.21**	0.38	0.38
Cidade de Xai Xai (Gaza)						
* An. funestus s.l*	0:**0.44**	0^b^	0^b^	0.09:0.09	0^b^	0^b^
* An. gambiae s.l*	–	–	–	**0.09**:0	5.56	5.56
* An. tenebrosus*	**3.78**:0.56	0^b^	0^b^	0.45:**2.18**	0^b^	0^b^
* An. ziemanni*	**1.00**:0.22	0^b^	0^b^	0.09:**0.36**	0	0.38
* An. pharoensis*	0:0	0^c^	0^c^	–	–	–
Cidade de Imhambane (Inhambane)						
* An. funestus s.l*	**1.38**:1.31	0.44	0.54	**1.38**:0.62	0.40	0.57
* An. gambiae s.l*	0.12:**0.19**	0^b^	0^b^	0:**0.13**	0^b^	0^b^
* An. tenebrosus*	0.06:**0.44**	0^b^	0^b^	0:**0.88**	0^b^	0^b^
* An. ziemanni*	0:**0.12**	0^b^	0^b^	0:**0.50**	0^b^	0^b^
Jangamo (Inhambane)						
* An. funestus s.l*	**6.31**:0	0.22	0.54	**1.71**:0.93	0.46	1.06
* An. gambiae s.l*	**0.89**:0	0.33	0.33	–	–	–
* An. tenebrosus*	0:0	0^c^	0^c^	–	–	–
Massinga (Inhambane)						
* An. funestus s.l*	**1.89**:1.44	0.32	0.32	**0.87**:0.47	1.07	1.59
* An. tenebrosus*	–	–	–	0:**0.20**	0^b^	0^b^
* An. ziemanni*	–	–	–	0:**0.13**	0^b^	0^b^

ND means ‘not determined’; – signifies zero mosquitoes in any of the collection methods

^a^highest value highlighted in bold

^b^feeding indoors and/or outdoors, but not found resting and/or exiting

^c^resting and/or exiting but not found feeding indoors and/or outdoors

### Chokwe district

*Anopheles gambiae s.l.* was the most abundant species during the rainy season and was found biting but not resting indoors (Fig. 3B). As such, the minimum IRS efficacy is zero, while the maximum IRS efficacy (again including exiting behaviour) was low (0.18) for this species (Table 2). A similar pattern was observed for *An. pharoensis* and *An. ziemanni*. Both *An. funestus s.l.* and *An. tenebrosus* were observed to rest indoors, resulting in minimum IRS efficacies of 0.12 and 0.05, respectively. Larger numbers of both species were observed in the window exit traps, leading to maximum IRS efficacies of 0.91 and 0.93, respectively.

During the dry season, all species above were also found biting outdoors and all, except for *An. funestus s.l.*, showed a higher tendency to feed outdoors. The number of indoor resting mosquitoes increased for all species (but remained zero for *An. gambiae s.l.*), compared to the rainy season, which lead to increased minimum IRS efficacies (ranging from 0.14 for *An. tenebrosus* to 2.50 for *An. funestus s.l.*).

### Cidade de Xai-Xai

During the rainy season, *An. pharoensis* was found resting indoors in a single house. No other mosquitoes were found resting inside nor exiting houses during the night (Fig. 3C). *Anopheles pharoensis* was not found in the human-baited tent traps, but several other species were captured. *Anopheles funestus s.l.* was more exophilic, whereas both *An. tenebrosus* and *An. ziemanni* were more endophilic. Combined, this leads to minimum and maximum IRS efficacies of zero, albeit *An. pharoensis* rested indoors (Table 2).

During the dry season, only *An. gambiae s.l.* was found resting indoors, and only *An. ziemanni* was observed in exit trap collections, which leads to a minimum and maximum IRS efficacy of 5.56 for *An. gambiae s.l.* and a maximum IRS efficacy of 0.38 for *An. ziemanni*. Of note is that a few *An. funestus s.l.* and larger numbers of *An. tenebrosus* were showing both endo- and exophilic behaviours, but as no individuals were found resting indoors and/or exiting houses, IRS is expected to be not effective against those vector species (both minimum and maximum IRS efficacy are zero).

### Cidade de Inhambane

During both seasons, *An. funestus s.l.* was the only species found resting indoors and leaving the house during the night (Fig. 3D). It was also the main species found biting indoors, although endophilic *An. gambiae s.l.* and *An. tenebrosus* were observed as well. The minimum IRS efficacy for *An. funestus s.l.* was 0.44 and 0.40 for the rainy and dry season, respectively, and the maximum IRS efficacy 0.54 and 0.57, respectively (Table 2). The IRS efficacy values for all other species was zero for both seasons.

Whereas *An. funestus* was typically endophilic in its behaviour, three other species (*An. gambiae s.l.*, *An. tenebrosus* and *An. ziemanni*) were more exophilic.

### Jangamo district

During both seasons, *An. funestus s.l.* was the main species found feeding and resting indoors, leaving the house during the night as well as feeding outdoors (Fig. 3E). The minimum IRS efficacy for *An. funestus s.l.* was 0.22 and 0.46 for the rainy and dry season, respectively, and the maximum IRS efficacy 0.54 and 1.06, respectively (Table 2). *Anopheles gambiae s.l.* was only observed to rest and feed indoors during the rainy season, but in lower numbers than *An. funestus s.l.* The minimum and maximum IRS efficacy for this species were 0.33.

### Massinga district

During both seasons, *An. funestus s.l.* was the main species found feeding and resting indoors, leaving the house during the night as well as feeding outdoors (Fig. 3F). The minimum IRS efficacy for *An. funestus s.l.* was 0.32 and 1.07 for the rainy and dry season, respectively, and the maximum IRS efficacy 0.32 and 1.59, respectively (Table 2). Several individuals of both *An. tenebrosus* and *An. ziemanni* showed exophilic behaviour during the dry season but were not encountered indoors, leading to estimated IRS efficacies of zero.

### Insecticide susceptibility tests

*Anopheles funestus s.l.* was susceptible to pirimiphos-methyl in two of the districts tested (Jangamo and Bilene), but susceptibility needs to be confirmed for Massinga (where 97% mortality was observed). Resistance to deltamethrin was detected in the Jangamo district (Table 3). No molecular species identification was performed on the *Anopheles funestus s.l.* individuals used in the susceptibility assays.

**Table 3:** Insecticide susceptibility status of *Anopheles funestus s.l.* in the districts of Bilene (Gaza Province), Massinga (Inhambane Province) and Jangamo (Inhambane Province) in 2018

Insecticide	Bilene		Jangamo		Massinga	
	Exposed	Control	Exposed	Control	Exposed	Control
Deltamethrin 0.05%	–	–	80% (30)	0% (19)	100% (10)	0% (11)
Pirimiphos-methyl 0.25%	100% (70)	7% (56)	100% (26)	5% (20)	97% (33)	0% (38)

## Discussion

This study was designed to test a novel, evidence-based SnES approach, with the aim to generate timely, informative and actionable data to answer the following programmatic question: ‘Will indoor residual spraying be effective in currently untargeted areas in Gaza and Inhambane provinces?’ The ESPT [15] was utilized to develop the targeted sampling framework using minimal essential indicators in an operationally relevant and feasible manner.

To answer the programmatic question of ‘Will indoor residual spraying be effective in currently untargeted areas in Gaza and Inhambane provinces?,’ data indicate that, based on the overlap of how IRS functions (on indoor resting and insecticide susceptible behaviours) and local *Anopheles* vector behaviours (the extent to which vectors enter houses, rest on walls and are susceptible to an IRS insecticide), IRS would be an effective intervention for *Anopheles funestus,* the primary documented vector resting indoors, using an insecticide for which the vector demonstrates susceptibility. Data also indicate that—from an entomological perspective—an IRS-based ‘one-size-fits-all’ vector control approach is unlikely to be effective in southern Mozambique’s malaria elimination strategy since vectors also function outside the scope of IRS functionality—being both exophagic and exophilic. *Anopheles funestus*, with its endophilic and endophagic bionomic traits, may be more effectively controlled by IRS (with the appropriate insecticide), while other species may be less affected. Note that even exophilic and exophagic mosquitoes that show some degree of indoor resting will be impacted by IRS [26].

When looking at specific geographies, in Bilene (Gaza province), Jangamo (Inhambane province) and Massinga (Inhambane province), *An. funestus* was the major vector species, biting and resting indoors, suggesting that IRS is an appropriate vector control intervention in these geographies to reduce malaria transmission. In other districts (cidade de Xai-Xai and -to a lesser extent- cidade de Inhambane in Inhambane province), IRS may have some impact on malaria transmission, given the fact that *An. funestus* is found resting indoors, but the presence of other vector species both indoors (biting, not resting) and outdoors suggests that alternative vector control interventions that target these gaps in protection may be needed to achieve the NMCP’s goals of elimination. Note that this data, along with epidemiological data, resulted in the NMCP and partners targeting IRS to a subset of districts in Gaza and Inhambane provinces in August 2019. In Gaza Province, Bilene and Limpopo districts were sprayed, and in Inhambane province C. de Inhambane and Maxixe (a district on the other side of the Inhambane Bay from cidade de Inhambane). Continued entomology surveillance, combined with epidemiological data, will allow for an operational evaluation of the efficacy of IRS targeting in subsequent years.

A secondary objective of this study was to evaluate the use of PSCs in understanding species-specific bionomic traits. PSC data alone are limited both by the behaviours PSCs are able to capture (only mosquito specimens that rest indoors) and by time (in the morning when PSCs are performed). Based on the range of collection methods used in the present study, results highlight that PSC data alone (performed in most of Mozambique’s entomological sentinel sites at the time of the study) when deciding to perform IRS may not be appropriate for understanding drivers of transmission as well as decision making given the overall range of *Anopheles* species present and their bionomic traits. PSCs do not collect data on biting behaviours (as a proxy for exposure to malaria), are unable to determine overall mosquito compositions (e.g. those that do not come indoors), and do not capture mosquitoes that leave the house prior to the time the PSC is conducted. Here, whilst PSCs identified *An. funestus* as the main *Anopheles* mosquito resting indoors in all districts except for in Chokwe and cidade de Xai-Xai, other surveillance tools demonstrated the presence of several other potential malaria vector species in all surveilled districts. PSCs may fail to capture important vectors, thereby neglecting to capture changing drivers of transmission and limiting a more complete understanding of the transmission system towards optimal decision making, including the use of LLINs as the primary vector control strategy in Mozambique. The use of a question-based approach—specifically catering the sampling methods to the question—enables directed and focused entomological intelligence for decision-making [15].

Other key findings are that *An. funestus s.s*. and *An. tenebrosus* were incriminated as vectors of *P. falciparum*. This confirms data that has demonstrated *An. funestus s.s*. has been a major malaria vector in southern Mozambique [27–29]. In 1999, low optical density (OD) in ELISAs suggested that *An. tenebrosus* may be a malaria vector in this geography [27]. In addition, the introduction of a boiling step in the present study to eliminate false positives [21] improved ELISA specificity and the strong OD values confer the vector status of *An. tenebrosus*. This species, as well as *An. ziemanni,* both members of the *An. coustani* group [30], were both identified in this study. Having said that, further molecular identification of *An. tenebrosus* individuals is warranted, as in Mopeia District (Zambezia Province, central Mozambique) individuals that were identified morphologically as *An. tenebrosus* were subsequently identified as *Anopheles namibiensis* based ITS2 gene sequencing results [31]. Although no ELISA positive mosquitoes from the *An. gambiae* complex (primary vectors) were reported, both *An. arabiensis* and *An. merus* are known to transmit malaria in southern Mozambique [5, 32] and may therefore still play a role in the local malaria transmission. Even though secondary vectors found in this study were not found to be malaria-positive, species such as *An. pharoensis* are also known malaria vectors in sub-Saharan Africa [33]. Though two species have been confirmed as vectors in this study, the possibility that other species also contribute to transmission remains a possibility when factoring in the SnES-based sampling frame.

When looking at *Anopheles* bionomic traits, as expected, *An. funestus s.l.* was found to be both endophagic (HBTT data) and endophilic (PSC data) (Additional file 2: Table S2 and Additional file 3: Table S3). Data from all districts indicate that outdoor biting occurs as well. *Anopheles funestus s.l.* was found to rest indoors at all sites, a proportion of which were found leaving before the PSC time point from WTs. It remains to be determined if these mosquitoes leaving the structure prior to the morning PSCs rested on the walls for a period. This is critical information if we are to accurately estimate the maximum IRS efficacy for any vector species in a particular location.

*Anopheles gambiae* complex were found host-seeking both indoors and outdoors, with *An. arabiensis*, *An. merus* and *An. quadriannulatus* having higher capturing densities outdoors, which is typical for this species [34, 35]. Interestingly, *An. quadriannulatus*, a typically zoophagic species [36], was found host-seeking in the human-baited trap and this unexpected anthropophilic behaviour could make it a modest vector [37, 38]. *Anopheles arabiensis* was the only member of this species complex found resting indoors in low numbers in the morning, but data indicate that all members were found exiting houses (WT data from Chokwe)—indicating house entry with undetermined resting behaviour prior to the morning time point of PSCs. Hourly indoor aspirations throughout the night would enable the evaluation of any resting behaviour towards understanding the potential impact of IRS.

Susceptibility to pirimiphos-methyl was found in the three districts included, indicating an organophosphate IRS product will effectively kill susceptible mosquitoes when resting on treated wall surfaces. However, collecting wild mosquitoes and using them directly in WHO tube bioassays to assess their insecticide susceptibility status is not the preferred method, as those mosquitoes will differ in their physiological age and feeding status [17]. As this method does allow for results within the SnES sampling week (in comparison to collecting blood-fed females or larvae from the field, and rear those to the next adult generation prior to testing), it will be critical to evaluate the difference in insecticide susceptibility outcomes between the different mosquito collection methods. Resistance to deltamethrin was detected Jangamo district. This latter outcome is not unique as pyrethroid resistance in *An. funestus s.l.* is common in southern Mozambique [39, 40], and the responsible allele seems to be fixed in the mosquito populations, which may even lead to a loss in the efficacy of PBO-pyrethroid LLINs [41]. The effectiveness of this net type of net is currently being evaluated in northern Mozambique.

There are several limitations to this operational surveillance study directed at decision making. Entomological collections were performed at the end of the rainy season and the beginning of the dry season, whilst IRS is normally implemented before the onset of and into the rainy season (August to December). Ideally, this snapshot would represent transmission dynamics present when IRS is implemented—however, the data and implications are very relevant to decision making. Data collected demonstrated expected and substantial heterogeneity across indicators—including species composition, and biting and resting behaviours—in the six districts, with values differing between sampling periods (approximately 8 week difference representing the wet and dry seasons). Data heterogeneity may be attributed to the limited number of sampling days associated with the ‘snapshot’ approach, typical or normal entomological surveillance and normal variation in drivers of mosquito populations including interventions, topography, land use, climate, human population densities, and connectivity, amongst other factors [27, 31, 42–45]. As such, obtaining estimates of the IRS efficacy indicators during the months when IRS is typically implemented would be valuable. Apart from this temporal scale, the observed heterogeneity in species diversity and densities between sites also strengthens the idea that collecting entomological intelligence on smaller spatial scales can result in a more targeted vector control approach [45, 46].

The HBTT utilized in this study demonstrates the adaptive implementation of a sampling tool based on local circumstances towards answering a specific question. The presence of *Aedes aegypti* and *Aedes albopictus* [23, 47] associated with outbreaks of Dengue virus in Mozambique [48], resulted in the risk-assessment based termination of HLC, considered the gold standard in vector surveillance. Though the HBTTs functioned well to capture *Anopheles*, there remains the inability to directly compare them to HLCs in order to understand true landing rates. This sampling tool may also require modifications as the tent, when placed outdoors, may be perceived as a structure by a mosquito (and capture may be considered to be based on “indoor” entry), and when placed indoors (towards standardization with outdoor catches) the tent adds an extra boundary for the mosquito to cross—thereby possibly reducing capture rates in both spaces.

Estimates of the maximum IRS efficacy can be improved by recording the feeding status of the mosquitoes collected with the window exit trap method. If mosquitoes are newly fully fed or exit the house unfed, one could assume they did not come in contact with indoor wall surfaces, but are more likely to rest outdoors or look for another blood meal, respectively. This mosquito cohort may be excluded when calculating the maximum IRS efficacy. In addition, estimates of both the minimum and the maximum possible impact of IRS are expected to be impacted by the insecticide resistance status of the vectors to the IRS product(s), but as the relationship between IRS efficacy and vector susceptibility status is unknown (and will depend on e.g., the active ingredient and its bio-availability, vector species and its contact time), the minimum and maximum possible impact of IRS are estimates for susceptible mosquitoes. Moreover, the resistance status is typically only quantified for the major vector species (*An. gambiae s.l.* and *An. funestus s.l.* in southern Mozambique), and not for other species that are found resting indoors (such as *An. pharoensis* and *An. tenebrosus* in the present study). Assessing the susceptibility of all (potential) vector species to the several insecticides that can be used in IRS programmes may be difficult in low transmission areas, given the low densities of vector populations (often due to scaled-up vector control efforts), but will be valuable to understand the impact of IRS on all those species.

Recent efforts towards sub-national malaria elimination in southern Mozambique with associated overall reductions in transmission may reduce the applicability of both vector incrimination and using EIR as an informative entomological endpoint. The usefulness of these indicators in low transmission continuum settings need to be re-evaluated as any output may not be representative of transmission due to lower densities of vector populations, and HLC-proxy sampling methods used may not be indicative of biting rates. The presence of multiple vectors with varying bionomic traits both increases the temporal and spatial nature of exposure as well as limits the efficacy of any single vector control tool.

A single ELISA positive mosquito, morphologically identified as an *An. funestus s.l.*, failed to be identified by PCR, together with other 191 *An. funestus s.l.* mosquitoes. Possible reasons may include morphological misidentification, DNA degradation, or the presence of another member of the *An. funestus* species complex not incorporated into the *An. funestus* complex PCR diagnostic [20]. There also exists the possibility of a novel species that were identified as *An. funestus* as reported in other east African geographies [49, 50].

Finally, epidemiological data indicate that *Plasmodium malariae* and *Plasmodium ovale* consist of up to 9% and 1% of diagnosed infections, respectively, with *P. falciparum* responsible for the rest [16]. Consequently, ELISAs used may have also underestimated *Plasmodium* infection rates since they were limited to detecting *P. falciparum* and *P. vivax* only, and did not include other parasite species known to circulate in Mozambique.

In conclusion, the SnES and ESPT-based approach implemented in six different districts in Gaza and Inhambane provinces was successful in producing targeted and focused data with an impact on decision-making for IRS targeting. The approach presented here may be adapted by using suitable entomological indicators and sampling methods to answer other programmatic questions; plan entomological surveillance activities for baseline, routine, foci, or outbreak surveys; and guide vector control targeting and tailoring for malaria control and elimination.

## Supplementary Information

**Additional file 1:** **Table S1.** Dates on which each sentinel site was visited.

**Additional file 2:** **Table S2.** Mean numbers of mosquitoes collected per person (with 95% CI), shown for each geography (district), season (rainy versus dry) and anopheline species.

**Additional file 3:** **Table S3.** Mean numbers of mosquitoes (standard deviation, SD) collected per room, shown for each geography (district), season (rainy versus dry) and anopheline species.

## Data Availability

The data supporting the conclusions of this manuscript are available in the tables in the main text as well as the supplementary tables.
