# Supplementary material for: To spray or target mosquitoes another way: focused entomological intelligence guides the implementation of indoor residual spraying in southern Mozambique
Source: Malar J. 2022 Jul 10;21:215. doi: 10.1186/s12936-022-04233-3 (PMC9275269; doi:10.1186/s12936-022-04233-3)
Supplement: Supplementary file 1 — Additional file 1: Table S1. Dates on which each sentinel site was visited. [file 12936_2022_4233_MOESM1_ESM.docx]

**Additional Table S1** | Dates on which each sentinel site was visited

| **Sentinel Site (Province)** | **Rainy season** | **Dry season** |
| --- | --- | --- |
| Bilene (Gaza) | 08 to 10 May, 2018 | 12 to 15 June, 2018 |
| Chokwe (Gaza) | 17 to 20 April, 2018 | 10 to 13 July, 2018 |
| Cidade de Xai-Xai (Gaza) | 22 to 25 May, 2018 | 27 to 29 June, 2018 |
| Cidade de Inhambane (Inhambane) | 05 to 08 June, 2018 | 24 to 26 July, 2018 |
| Jangamo (Inhambane) | 24 to 27 April, 2018 | 03 to 06 July, 2018 |
| Massinga (Inhambane) | 15 to 18 May, 2018 | 19 to 22 June, 2018 |
