# Supplementary material for: To spray or target mosquitoes another way: focused entomological intelligence guides the implementation of indoor residual spraying in southern Mozambique
Source: Malar J. 2022 Jul 10;21:215. doi: 10.1186/s12936-022-04233-3 (PMC9275269; doi:10.1186/s12936-022-04233-3)
Supplement: Supplementary file 2 — Additional file 2: Table S2. Mean numbers of mosquitoes collected per person (with 95% CI), shown for each geography (district), season (rainy versus dry) and anopheline species. [file 12936_2022_4233_MOESM2_ESM.docx]

**Additional Table S2. Mean numbers of mosquitoes collected per person (with 95% CI), shown for each geography (district), season (rainy versus dry) and anopheline species.**

|  | **Resting** | **Exiting** | **Feeding indoor** | **Feeding outdoor** |
| --- | --- | --- | --- | --- |
| ***Bilene (Gaza) - Rainy season*** | | | | |
| *An. funestus* *s.l.* | 5.16 (2.2 – 8.1) | 2.24 (-0.35 – 4.82) | ND | ND |
| *An. gambiae* *s.l.* | 0.09 (-0.04 – 0.21) | -- | ND | ND |
| ***Bilene (Gaza) - Dry season*** | | | | |
| *An. funestus* *s.l.* | 4.06 (1.54 – 6.59) | 4.44 (-1.1 – 9.97) | 4.69 (0.64 – 8.74) | 0.77 (-0.04 – 1.57) |
| *An. gambiae* *s.l.* | 0.11 (-0.11 – 0.33) | 0.09 (-0.04 – 0.22) | -- | 0.31 (0.05 – 0.57) |
| *An. ziemanni* | -- | -- | 0.08 (-0.07 – 0.23) | 0.08 (-0.07 – 0.23) |
| ***Chokwe (Gaza) - Rainy season*** | | | | |
| *An. funestus s.l.* | 0.09 (-0.03 – 0.2) | 0.59 (0.28 – 0.9) | 0.75 (0.23 – 1.27) | -- |
| *An. tenebrosus* | 0.04 (-0.04 – 0.123) | 0.64 (0.06 – 1.2) | 0.73 (0.13 – 1.32) | -- |
| *An. gambiae s.l.* | -- | 0.23 (-0.005 – 0.46) | 1.30 (0.25 – 2.35) | -- |
| *An. pharoensis* | -- | 0.03 (-0.03 – 0.1) | 0.18 (-0.17 – 0.54) | -- |
| *An. ziemanni* | -- | 0.07 (0.026 – 0.124) | 0.48 (-0.07 – 1.02) | -- |
| ***Chokwe (Gaza) - Dry season*** | | | | |
| *An. funestus s.l.* | 0.65 (-0.15 – 1.45) | 0.08 (-0.02 – 0.17) | 0.21 (0.02 – 0.4) | 0.05 (-0.05 – 0.16) |
| *An. pharoensis* | 0.04 (-0.04 – 0.12) | -- | -- | 0.11 (-0.04 – 0.25) |
| *An. tenebrosus* | 0.15 (0.002 – 0.31) | 0.08 (-0.02 – 0.17) | 0.42 (0.15 – 0.69) | 0.68 (-0.02 – 1.39) |
| *An. ziemanni* | 0.14 (-0.14 – 0.42) | -- | 0.16 (-0.01 – 0.33) | 0.21 (-0.03 – 0.45) |
| *An. gambiae s.l.* | -- | 0.04 (-0.04 – 0.12) | 0.11 (-0.1 – 0.31) | 0.58 (-0.01 – 1.17) |
| ***Cidade de Xai-Xai (Gaza) - Rainy season*** | | | | |
| *An. funestus s.l.* | -- | -- | -- | 0.44 (-0.22 – 1.11) |
| *An. pharoensis* | 0.25 (0*) | -- | -- | -- |
| *An. tenebrosus* | -- | -- | 3.78 (-3.14 – 10.7) | 0.56 (0.08 – 1.03) |
| *An. ziemanni* | -- | -- | 1.00 (-0.96 – 2.96) | 0.22 (-0.07 – 0.51) |
| ***Cidade de Xai-Xai (Gaza) - Dry season*** | | | | |
| *An. funestus s.l.* | -- | -- | 0.09 (-0.09 – 0.27) | 0.09 (-0.09 – 0.27) |
| *An. tenebrosus* | -- | -- | 0.45 (0.05 – 0.86) | 2.18 (0.92 – 3.44) |
| *An. ziemanni* | -- | 0.17 (-0.16 – 0.49) | 0.09 (-0.09 – 0.27) | 0.36 (-0.18 – 0.9) |
| *An. gambiae s.l.* | 0.50 (-0.48 – 1.48) | -- | 0.09 (-0.09 – 0.27) | -- |
| ***Cidade de Inhambane (Inhambane) - Rainy season*** | | | | |
| *An. funestus s.l.* | 1.18 (0.64 – 1.72) | 0.26 (0.08 – 0.45) | 1.38 (0.3 – 2.45) | 1.31 (0.11 – 2.52) |
| *An. pharoensis* | -- | -- | -- | -- |
| *An. tenebrosus* | -- | -- | 0.06 (-0.06 – 0.19) | 0.44 (-0.03 – 0.91) |
| *An. ziemanni* | -- | -- | -- | 0.12 (-0.04 – 0.29) |
| *An. gambiae s.l.* | -- | -- | 0.12 (-0.04 – 0.29) | 0.19 (-0.18 – 0.56) |
| ***Cidade de Inhambane (Inhambane) - Dry season*** | | | | |
| *An. funestus s.l.* | 0.8 (0.3 – 1.28) | 0.33 (0.08 – 0.59) | 1.38 (-0.24 – 2.99) | 0.62 (0.11 – 1.14) |
| *An. tenebrosus* | -- | -- | -- | 0.88 (-0.14 – 1.89) |
| *An. ziemanni* | -- | -- | -- | 0.50 (-0.24 – 1.24) |
| *An. gambiae s.l.* | -- | -- | -- | 0.13 (-0.12 – 0.37) |
| ***Jangamo (Inhambane) - Rainy season*** | | | | |
| *An. funestus s.l.* | 1.40 (0.34 – 2.45) | 2.02 (0.7 – 3.34) | 6.31 (2.34 – 10.27) | -- |
| *An. tenebrosus* | -- | 0.04 (-0.04 – 0.12) | -- | -- |
| *An. gambiae s.l.* | 0.29 (-0.19 – 0.78) | -- | 0.89 (-0.11 – 1.89) | -- |
| ***Jangamo (Inhambane) - Dry season*** | | | | |
| *An. funestus s.l.* | 1.21 (0.32 – 2.1) | 1.59 (0.95 – 2.22) | 1.71 (0.51 – 2.92) | 0.93 (0.26 – 1.59) |
| ***Massinga (Inhambane) - Rainy season*** | | | | |
| *An. funestus s.l.* | 1.07 (0.46 – 1.67) | -- | 1.89 (0.31 – 3.47) | 1.44 (0.09 – 2.8) |
| ***Massinga (Inhambane) – Dry season*** | | | | |
| *An. funestus s.l.* | 1.43 (0.21 – 2.64) | 0.70 (0.09 – 1.31) | 0.87 (0.27 – 1.47) | 0.47 (-0.1 – 1.04) |
| *An. tenebrosus* | -- | -- | -- | 0.20 (-0.01 – 0.41) |
| *An. ziemanni* | -- | -- | -- | 0.13 (-0.04 – 0.31) |

-- signifies zero mosquitoes; ND means ‘not determined’; ^*^SD=0 because mosquitoes were collected from one house only
