# Supplementary material for: To spray or target mosquitoes another way: focused entomological intelligence guides the implementation of indoor residual spraying in southern Mozambique
Source: Malar J. 2022 Jul 10;21:215. doi: 10.1186/s12936-022-04233-3 (PMC9275269; doi:10.1186/s12936-022-04233-3)
Supplement: Supplementary file 3 — Additional file 3: Table S3. Mean numbers of mosquitoes (standard deviation, SD) collected per room, shown for each geography (district), season (rainy versus dry) and anopheline species. [file 12936_2022_4233_MOESM3_ESM.docx]

**Additional Table S3 Mean numbers of mosquitoes (standard deviation, SD) collected per room , shown for each geography (district), season (rainy versus dry) and anopheline species.**

|  | **Resting** | **Exiting** |
| --- | --- | --- |
| ***Bilene (Gaza) - Rainy season*** | | |
| *An. funestus* *s.l.* | 10.38 (8.90) | 23.50 (6.09) |
| *An. gambiae* *s.l.* | 0.25 (0.46) |  |
| ***Bilene (Gaza) - Dry season*** | | |
| *An. funestus* *s.l.* | 6.00 (5.07) | 6.67 (9.94) |
| *An. gambiae* *s.l.* | 0.11 (0.33) | 0.22 (0.44) |
| ***Chokwe (Gaza) - Rainy season*** | | |
| *An. funestus s.l.* | 0.33 (0.52) | 1.83 (0.98) |
| *An. tenebrosus* | 0.17 (0.41) | 2.50 (2.81) |
| *An. gambiae s.l.* |  | 0.83 (0.98) |
| *An. pharoensis* |  | 0.17 (0.41) |
| *An. ziemanni* |  | 0.33 (0.52) |
| ***Chokwe (Gaza) - Dry season*** | | |
| *An. funestus s.l.* | 1.14 (1.21) | 0.29 (0.49) |
| *An. pharoensis* | 0.14 (0.38) |  |
| *An. tenebrosus* | 0.43 (0.53) | 0.29 (0.49) |
| *An. ziemanni* | 0.14 (0.38) |  |
| *An. gambiae s.l.* |  | 0.14 (0.38) |
| ***Cidade de Xai-Xai (Gaza) - Rainy season*** | | |
| *An. pharoensis* | 1.00 (0*) |  |
| ***Cidade de Xai-Xai (Gaza) - Dry season*** | | |
| *An. ziemanni* |  | 0.50 (0.71) |
| *An. gambiae s.l.* | 0.50 (0.71) |  |
| ***Cidade de Inhambane (Inhambane) - Rainy season*** | | |
| *An. funestus s.l.* | 2.57 (1.72) | 0.57 (0.53) |
| ***Cidade de Inhambane (Inhambane) - Dry season*** | | |
| *An. funestus s.l.* | 2.14 (1.77) | 0.71 (0.49) |
| ***Jangamo (Inhambane) - Rainy season*** | | |
| *An. funestus s.l.* | 3.25 (3.15) | 4.00 (2.39) |
| *An. tenebrosus* |  | 0.12 (0.35) |
| *An. gambiae s.l.* | 0.62 (1.41) |  |
| ***Jangamo (Inhambane) - Dry season*** | | |
| *An. funestus s.l.* | 3.00 (3.04) | 4.00 (2.12) |
| ***Massinga (Inhambane) - Rainy season*** | | |
| *An. funestus s.l.* | 3.57 (3.15) |  |
| ***Massinga (Inhambane) - Dry season*** | | |
| *An. funestus s.l.* | 3.25 (4.20) | 1.25 (1.58) |

** SD = 0 because there is only 1 house*
